# Radiofrequency ablation versus stereotactic body radiotherapy for small hepatocellular carcinoma: a Markov model‐based analysis

**DOI:** 10.1002/cam4.893

**Published:** 2016-10-05

**Authors:** Young‐Seok Seo, Mi‐Sook Kim, Hyung‐Jun Yoo, Won Il Jang, Eun Kyung Paik, Chul Ju Han, Byung‐Hee Lee

**Affiliations:** ^1^ Department of Radiation OncologyKorea Institute of Radiological & Medical SciencesSeoulKorea; ^2^ Department of Internal MedicineKorea Institute of Radiological & Medical SciencesSeoulKorea; ^3^ Department of RadiologyKorea Institute of Radiological & Medical SciencesSeoulKorea

**Keywords:** Hepatocellular carcinoma, Markov, radiofrequency ablation, stereotactic

## Abstract

The aim of this study is to compare radiofrequency ablation (RFA) with stereotactic body radiotherapy (SBRT) for hepatocellular carcinomas (HCC) smaller than 3 cm. A Markov cohort model was developed to simulate a cohort of patients aged 60–65 years with small HCCs who had undergone either RFA or SBRT and were followed up over their remaining life expectancy. The inclusion criteria were: (1) HCC ≤3 cm in diameter with ≤ 3 nodules; (2) absence of extrahepatic metastasis or portal/hepatic vein invasion; (3) Child‐Pugh Class A or B. Twenty thousand virtual patients were randomly assigned to undergo RFA or SBRT. Predicted life expectancy was 6.452 and 6.371 years in the RFA and SBRT groups, respectively. The probability distributions of the expected overall survival were nearly identical. The 95% confidence intervals were 6.25–6.66 and 6.17–6.58 years for RFA and SBRT, respectively. The difference between RFA and SBRT was insignificant (*P* = 0.2884). Two‐way sensitivity analysis demonstrated that if the tumor is 2–3 cm, SBRT is the preferred treatment option. Our Markov model has shown that expected overall survival of SBRT is nearly identical to RFA in HCCs smaller than 3 cm, but SBRT may have an advantage for tumors 2 cm and larger. A randomized trial is required to confirm these findings.

## Introduction

Hepatocellular carcinoma (HCC) is the third leading cause of cancer‐related deaths globally 1. Diagnosis of HCC at an early stage enables the application of potentially curative treatments 2. Although surgery is the standard treatment, surgery is indicated in only 20–30% of HCC patients because liver cirrhosis is a contraindication for surgery 3, 4. Therefore, nonsurgical interventions have been explored, and radiofrequency ablation (RFA) has become the main treatment method for patients who are inoperable and have small HCCs 5, 6. RFA shows excellent outcomes with 70–90% local control for small HCCs 6, 7, 8, 9. Recently, stereotactic body radiation therapy (SBRT) has become an emerging noninvasive alternative to RFA with similar local control rates 10, 11, 12, 13. Wahl et al. reported that SBRT and RFA were equally effective in treating small HCCs 15.

Although research on SBRT for treating HCC has increased recently, there have been no randomized controlled trials comparing survival after SBRT and RFA. To investigate the effectiveness of SBRT as an alternative to RFA, prospective randomized controlled trials are necessary but it is difficult and very time consuming to conduct randomized controlled trials. A Markov model can conduct a computerized simulation that compares the outcomes of competitive treatment modalities, and identify major parameters influencing the results 15. Markov models are widely used in cost‐effectiveness analysis. Recently, several researchers have conducted virtual randomized trials via Markov models to compare the survival outcomes after RFA or surgery for HCC 15, 16.

In this study, we conducted a simulated randomized trial by Markov model analysis to compare the overall survival (OS) of patients with small HCCs after treatment with RFA or SBRT.

## Methods and Materials

### Computerized simulation

**Figure 1:**
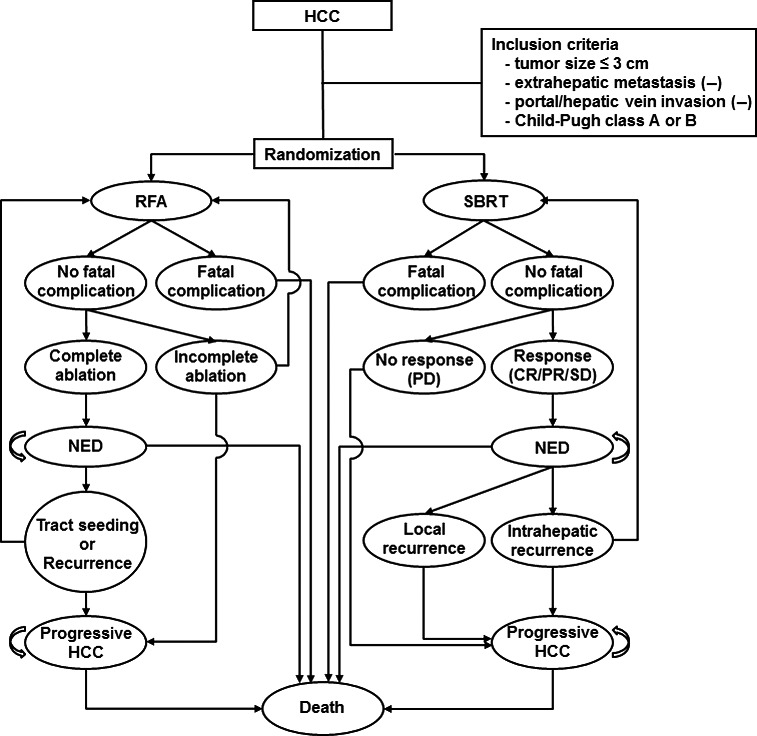
Scenario for the Markov state transition model of HCC carcinomas less than 3 cm. Each circle represents a state of health. From the initial state, patients are randomized to undergo RFA or SBRT. Straight arrows represent the changes that may occur during each cycle or a very short time interval. In contrast, circular arrows mean that the patients may remain in the same Markov state for more than one cycle. HCC, hepatocellular carcinoma; RFA, radiofrequency ablation; SBRT, stereotactic body radiotherapy; NED, no evidence of disease; CR, complete response; PR, partial response; SD, stable disease; PD, progressive disease.

A Markov model was developed to simulate a cohort of patients aged 60–65 years with small HCCs who had undergone either RFA or SBRT and were followed over a time horizon of their remaining life expectancy (Fig. 1). Inclusion criteria was as follows: (1) HCC ≤3 cm in diameter and no more than three tumor nodules; (2) absence of extrahepatic metastasis; (3) absence of portal/hepatic vein invasion; (4) Child‐Pugh Class A or B. Each virtual patient was randomly assigned to undergo RFA or SBRT and 10,000 patients were allocated to each group.

A Markov model was constructed with 18 health states, nine states for the RFA group and the remaining nine for the SBRT group. From the initial state, patients were randomized to undergo RFA or SBRT (Fig. 1). Patients could remain longer than one cycle in only two Markov states, which were no evidence of disease and a progressive HCC state, respectively. The transition probability from one state to another was determined according to values extracted from the literature (Table 1). The length cycle in the model was defined as 1 year and the Markov cycle was assumed to be repeated for 15 cycles because cirrhotic patients rarely survive for more than 15 years 16. The Markov model was generated with TreeAge Pro (TreeAge Software, Williamstown, MA). The annual mortality rates from the median survival of published Kaplan–Meier curves were calculated using the declining exponential approximation of life expectancy 17.

**Table 1:** Estimated values of the variables used for the Markov model extracted from the literature

Variables	RFA		SBRT
Annual mortality rate of general population (60–65 years old)		0.04985 [22]	
Annual mortality rate of cirrhotic patients		0.0221 [24]	
Annual mortality rate for progressive HCC	0.4498 (range, 0.3301–0.4498) [25, 26]		
Probability of procedure‐related mortality	0.006 [40]		0.0075 (0–0.011) [11‐13, 41]
Probability of initial tumor control failure	0.0418 (0.0333–0.05) [6, 18, 19]		0.0059 (0–0.021) [10, 12, 42]
Probability of needle tract seeding during RFA	0.0197 (0.0087–0.028) [7, 8, 43]		(‐)
Probability of local recurrence within 1 year following initial treatment	1–3 cm: 0.0567 (0.0085–0.1131) [6, 7‐9]2–3 cm: 0.2109 (0.1306–0.22) [6, 20, 34]		1–3cm: 0.0229 (0–0.0309) [10‐13]2–3cm: 0.0541 [12]
Probability of intrahepatic recurrence within 5 years		0.7 [44, 45]	
Probability of performing retreatment for recurrent HCC	0.65 (0.63–0.7931) [6, 20, 21]		
Maximum number of retreatments for recurrent HCC with same procedure	3 [28, 29]		2 (1–6) [30, 31]

HCC, hepatocellular carcinoma; RFA, radiofrequency ablation; SBRT, stereotactic body radiotherapy

In the RFA group, patients were evaluated by computed tomography scan 1 month after the end of the first cycle and, if there were incompletely ablated lesions, were treated once again by RFA 8, 9, 18. However, patients who did not respond to the second cycle of RFA for the incomplete ablation directly entered into the state of progressive HCC. In the SBRT group, treatment responses were evaluated 3–6 months after SBRT and patients with progressive disease directly entered the state of progressive HCC without repeating SBRT for the incompletely ablated lesion (Fig. 1). In the RFA group, some patients with local, intrahepatic recurrences, or needle tract seeding were treated with repeated RFA and others directly entered the state of progressive HCC without repeating RFA. In the SBRT group, some patients with intrahepatic recurrence were treated with repeated SBRT but all patients with local recurrence directly entered the state of progressive HCC without repeating SBRT.

To explore the best treatment strategy for OS, sensitivity analyses were conducted using a range of values for a single variable (one‐way sensitivity analysis) or across ranges of values for two or three variables simultaneously (multi‐way sensitivity analysis). A second‐order Monte Carlo probabilistic sensitivity analysis was carried out to evaluate the impact of parameter uncertainties on the model results.

### Systematic review for parameter estimation

An online database (MEDLINE on PubMed) was searched for abstracts or full articles in English from 1990 to September 2015. Terms used in our search included: “hepatocellular carcinoma,” “liver cancer,” or “primary liver carcinoma” as common text words combined with “stereotactic radiotherapy” or “radiofrequency ablation.” All estimated parameters used in this Markov model were obtained by a systematic review of the literature (Table 1). The parameters were preferentially extracted from randomized studies and, if that proved impossible, from prospective cohorts or retrospective cohort studies in the above order.

### Summary of parameters and assumptions

The mean age of patients in the cohort of this study was assumed to be 60–65 years considering the range of ages in the selected articles 6, 7, 9, 18, 19, 20, 21.

The annual mortality rate was estimated as the sum of two components, the annual mortality rate of the general population 22 and that of cirrhotic patients. We assumed that half of cirrhotic patients would die of cancer 23; therefore, the liver‐related mortality rate for compensated cirrhotic patients without cancer was estimated as 0.011 from 0.022 of the best report 23, 24. The median survival time for patients with progressive HCC is 1.16 years with a mortality rate of 0.4498 per year 25, 26. The expected 5‐year intrahepatic recurrence rate is at least 70% 27, and a declining exponential approximation was used to estimate the annual incidence of recurrence.

During follow‐up, patients with intrahepatic recurrence were considered candidates for retreatment with the same modalities. In the literature, 63–79% of patients with recurrent HCC who had been treated with RFA for the primary HCC were retreated with RFA again 6, 20, 21. However, the retreatment rates by SBRT for recurrent small HCCs have not been reported. Because the indication for retreatment for recurrent HCC would be similar in both the RFA and SBRT groups, we assumed the same probability of retreatment for recurrences in both groups. The probability of performing retreatment for local and intrahepatic recurrence or needle tract seeding was assumed to be identical to simplify the Markov model.

Many articles have reported that repeated RFA was completed safely for recurrent HCC 28, 29, 30. However, there was only one article that demonstrated the safety and efficacy of repeated SBRT for HCC 30. Lo et al. reported that repeated SBRT did not seem to affect liver function and is feasible with acceptable toxicity. The maximum number of repeated proton beam therapies for recurrent HCC is seven courses 31. Considering the reality in the clinical area, we set a limit for the maximum number of retreatments for recurrent HCC by each modality, as shown in Table 1.

We obtained data, which included the probability of procedure‐related mortality, failure of initial tumor control, needle tract seeding during RFA, and local control, from previously published studies. Because there was a paucity of data on outcomes of small HCCs (1–3 cm) with SBRT, we included data from several studies that treated HCCs smaller than 5 cm. To calculate an overall representative value for each component, each outcome in the average was weighed by the number of patients in the articles.

### Validation of Markov model

We evaluated the validity of our Markov model by comparing it with OS from previously reported studies that investigated HCC patients with tumors smaller than 3 cm. Predicted Kaplan–Meier survival curves and 95% confidence intervals (CI) from our Markov model were created and dots representing the survival outcomes of real studies were marked on the survival curves.

## Results

### Predicted life expectancy

**Figure 2:**
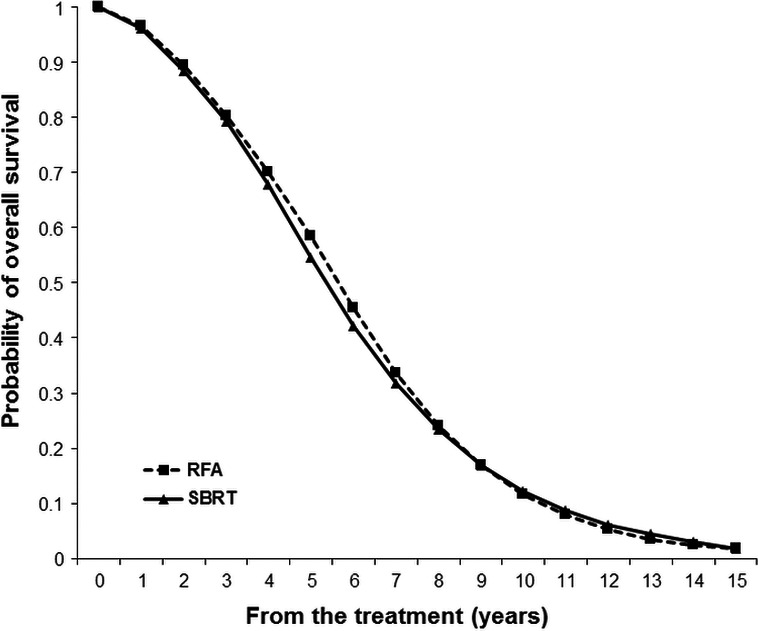
Expected overall survival curves of patients with small HCCs treated with RFA or SBRT. HCC, hepatocellular carcinoma; RFA, radiofrequency ablation; SBRT, stereotactic body radiotherapy.

For 60–65 year‐old patients, our model predicted a life expectancy of 6.452 and 6.371 years in the RFA and SBRT group, respectively. The expected 5‐year OS rates were calculated as 58.5% and 61.1% in the RFA and SBRT group, respectively (Fig. 2).

### Model validity

**Figure 3:**
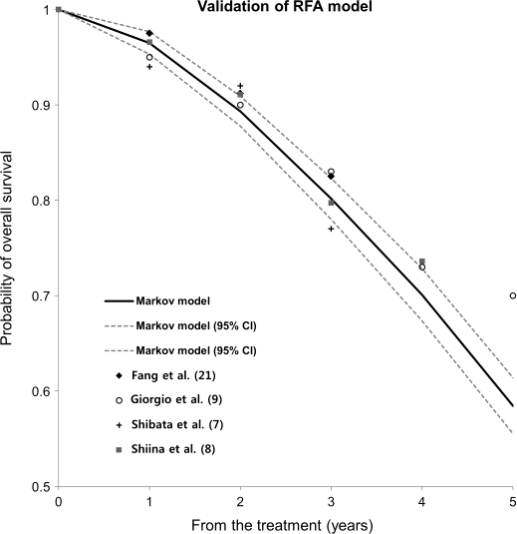
Validation of Markov model. Predicted survival curve after RFA and 95% confidence intervals (CI) from our Markov model is shown. The dots represent the survival outcomes of real studies that were marked on the survival curves. Almost all dots are positioned inside the 95% CI of the survival curve from the Markov model. CI, confidence interval; HCC, hepatocellular carcinoma; RFA, radiofrequency ablation.

Figure 3 illustrates the predicted survival rate after RFA. Outcomes from real randomized studies 7, 8, 9, 19 are marked by dots. The randomized studies have nearly the same survival outcomes as our cohort within the Markov model. Most dots are positioned inside the 95% CI of the survival curve from the Markov model. However, we could not validate the Markov model of SBRT because there was no real published study with SBRT that used the same cohort as our Markov model.

### One‐way sensitivity analysis

SBRT could be a preferred strategy if the probability of variables were changed beyond threshold (Table 2). Other variables did not change the preferred treatment option from RFA. The probability of intrahepatic recurrence or local recurrence after SBRT or RFA was the most important factor affecting OS outcomes in the tornado diagrams. However, treatment options like needle tract seeding during RFA or procedure‐related mortality had less influence on survival outcomes.

**Table 2:** One‐way sensitivity analysis: list of variables and respective threshold values influencing the overall survival from the Markov model

Variables	Threshold
Probability of local recurrence within 1 year after RFA	0.073
Probability of local recurrence within 1 year after SBRT	0.016
Probability of intrahepatic recurrence within 1 year after primary treatment	0.179
Maximum number of retreatments for recurrent HCC with RFA	2
Maximum number of retreatment for recurrent HCC with SBRT	3

RFA, radiofrequency ablation; SBRT, stereotactic body radiotherapy; HCC, hepatocellular carcinoma.

### Two‐way sensitivity analysis

**Figure 4:**
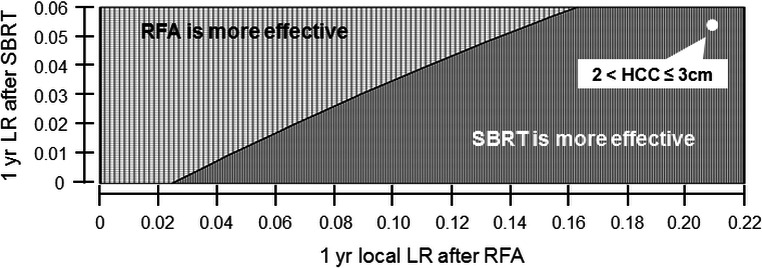
Two‐way sensitivity analysis: 1‐year local recurrence rate after RFA or SBRT. The overall survival of patients with a 1‐year LR of 1% after SBRT was very similar to the survival of those with 1 year LR of 2% after RFA when other variable values remained constant at preset values. If the tumor size was confined from 2 cm to 3 cm, 1 year LR was 0.2109 and 0.0541 for RFA and SBRT, respectively, and SBRT was the preferred treatment option (white dot in the figure). LR, local recurrence rate; RFA, radiofrequency ablation; SBRT, stereotactic body radiotherapy.

The OS of patients with a 1‐year local recurrence rate of 1% after SBRT was the same as that of patients with a 1‐year local recurrence rate of 2% after RFA, when other variable values were kept constant at predetermined values (Fig. 4). We stratified tumors by size. From the literature, tumor size correlates with the local recurrence rate for RFA and SBRT (Table 1). If the tumor size is confined from 2 cm to 3 cm, SBRT is the preferred treatment option (white dot in Fig. 4).

### Second‐order Monte Carlo simulation

The expected OS for RFA and SBRT were nearly the same as demonstrated in the probability distributions of OS for the cohort in this study (Figure S1, Figure S2). The 95% CIs were 6.25–6.66 and 6.17–6.58 years for RFA and SBRT, respectively. The 95% CI for the difference in OS between RFA and SBRT was −0.07 to 0.23 years (Figure S3). The difference between RFA and SBRT was insignificant (*P* = 0.2884).

## Discussion

In this study, we conducted a simulated randomized trial to compare the OS of patients with small HCCs treated with RFA or SBRT and we found that the OS outcomes of the two groups were nearly identical. Expected OS rates after RFA in the Markov model were comparable with that of the published literature 7, 8, 9, 19. In validation, the survival curve from the Markov model after RFA showed a very similar trend to the results from the published literature (Fig. 3). It seems reasonable, and reliable methods that predict OS indirectly using extracted parameters from previously reported literature such as local recurrence rates and procedure‐related mortality would be expected to accurately predict OS. However, we could not validate the Markov model of SBRT because real published studies had heterogeneous cohorts, including relatively large HCCs (over 3 cm) or recurrent HCC after primary treatment. On the other hand, in the Markov model, we assumed that patients treated with SBRT had small HCCs (<3 cm) and recurrent HCC cases were excluded. To validate the SBRT model properly, more studies that investigate patients with small HCCs (less than 3 cm) and naive to previous HCC treatment are necessary.

In the Markov model, the scenario of SBRT had disadvantages compared to RFA. First, patients with no response or local recurrence after SBRT directly entered the state of progressive HCC without repeating SBRT for the incompletely ablated lesion. Second, the maximum number of retreatments for recurrent HCC was limited to two courses of SBRT, whereas RFA could be performed three times. Third, the local recurrence rate following SBRT included data from several studies that treated HCCs smaller than 5 cm because of a paucity of data on outcomes of small HCCs (1–3 cm) treated with SBRT. Despite these disadvantages in the scenario, SBRT showed comparable OS.

In this study, SBRT was the preferred strategy compared to RFA if the tumor size was confined from 2 to 3 cm (Fig. 4). In the literature, for tumors smaller than 2 cm, RFA provides excellent local control that comes close to the outcomes with surgery 6, 20, 33. Livraghi et al. treated 218 patients with single HCCs <2.0 cm by RFA and reported a 0.9% local recurrence rate during follow‐up 33. However, the local recurrence rate was increased with HCC >2 cm (vs. those with HCC ≤2 cm) after RFA treatment 6, 14, 20, 34 due to increasing tumor tissue distance from the heat source and incomplete coagulative necrosis 6, 34. Lin et al. reported a 9% and 21% local recurrence rate for HCCs ≤2 cm and 2 cm < HCCs ≤3 cm, respectively, after RFA. In contrast, several studies previously reported that the local recurrence rate for SBRT was not greatly increased as the tumor size of HCC increased if the SBRT dose was sufficiently high 12, 14, 35.

There are several limitations to this study. First, parameters for SBRT were extracted from studies with heterogeneous tumor size (<5 cm) because of a lack of studies reporting outcomes when the tumor size was less than 3 cm. Although tumor size does not correlate with local control rates following SBRT, there is a possibility that the local control rate after SBRT has been underestimated. In practice, prescribing sufficiently high doses of SBRT is often restricted in large tumors after considering constraints of normal organs. Second, we simplified the scenario for convenience of handling with the Markov model. According to the scenario, patients with recurrence can only be treated with the same modality used for primary treatment (Fig. 1). However, in real clinical situations, RFA, SBRT, transarterial chemoembolization, percutaneous ethanol injection, or liver transplantation are available when recurrence has developed after primary treatment; in addition, sorafenib can be used to prolong the survival of patients with progressive HCC 27. Third, with regard to the fatal complications induced by each treatment modality in the scenario, it is possible that the parameter of the mortality rate following SBRT in this study does not represent the real mortality rate of small HCCs treated with SBRT. The mortality rate of SBRT used to treat small HCCs remains unclear because reports about complications of SBRT are insufficient yet as compared to RFA.

Applicability of both modalities for HCC is slightly different depending on the location in the liver and tumor size. If the tumor is located near the diaphragm, bowel, heart, bile ducts, or major vessels, or if the tumor size is over 3 cm, it is difficult to ablate the tumor with RFA 27. While the applicability of SBRT is relatively unconstrained compared to RFA in terms of tumor size and location; SBRT is also not applicable when the tumor is located near the bowel. SBRT is applicable for deep‐seated tumors in the liver that are inaccessible by RFA probes. Relatively large HCCs can be treated safely by SBRT if the normal liver volume (usually >700 cm^3^) is preserved sufficiently 36, 37. Also, hepatic toxicity ≥ grade 2 induced by SBRT does not generally lead to severe radiation‐induced morbidity or mortality in the long term 38. For example, Bae et al. reported that most patients with hepatic toxicity ≥ grade 2 did not experience further deterioration in hepatic function 39.

In conclusion, our Markov model has shown that expected OS after SBRT was nearly identical to RFA for treating small HCCs (less than 3 cm) and SBRT may have an advantage for tumors 2 cm and larger. These results suggest that SBRT could be considered an alternative to RFA for treating small HCCs. However, a randomized trial comparing SBRT to RFA for small HCCs is necessary to confirm our results and we hope that the results of this study can serve as the background and rationale for future prospective trials.

## Conflict of Interest

The authors declared no conflicts of interest in this study.

## Supporting information


**Figure S1**. Monte Carlo Probability Distribution: RFA
**Figure S2**. Monte Carlo Probability Distribution: SBRT
**Figure S3**. Incremental outcome: RFA vs. SBRT
