# Supplementary figures and images for: Radiofrequency ablation versus stereotactic body radiotherapy for small hepatocellular carcinoma: a Markov model‐based analysis
Source: Cancer Med. 2016 Oct 5;5(11):3094–101. doi: 10.1002/cam4.893 (PMC5119964; doi:10.1002/cam4.893)

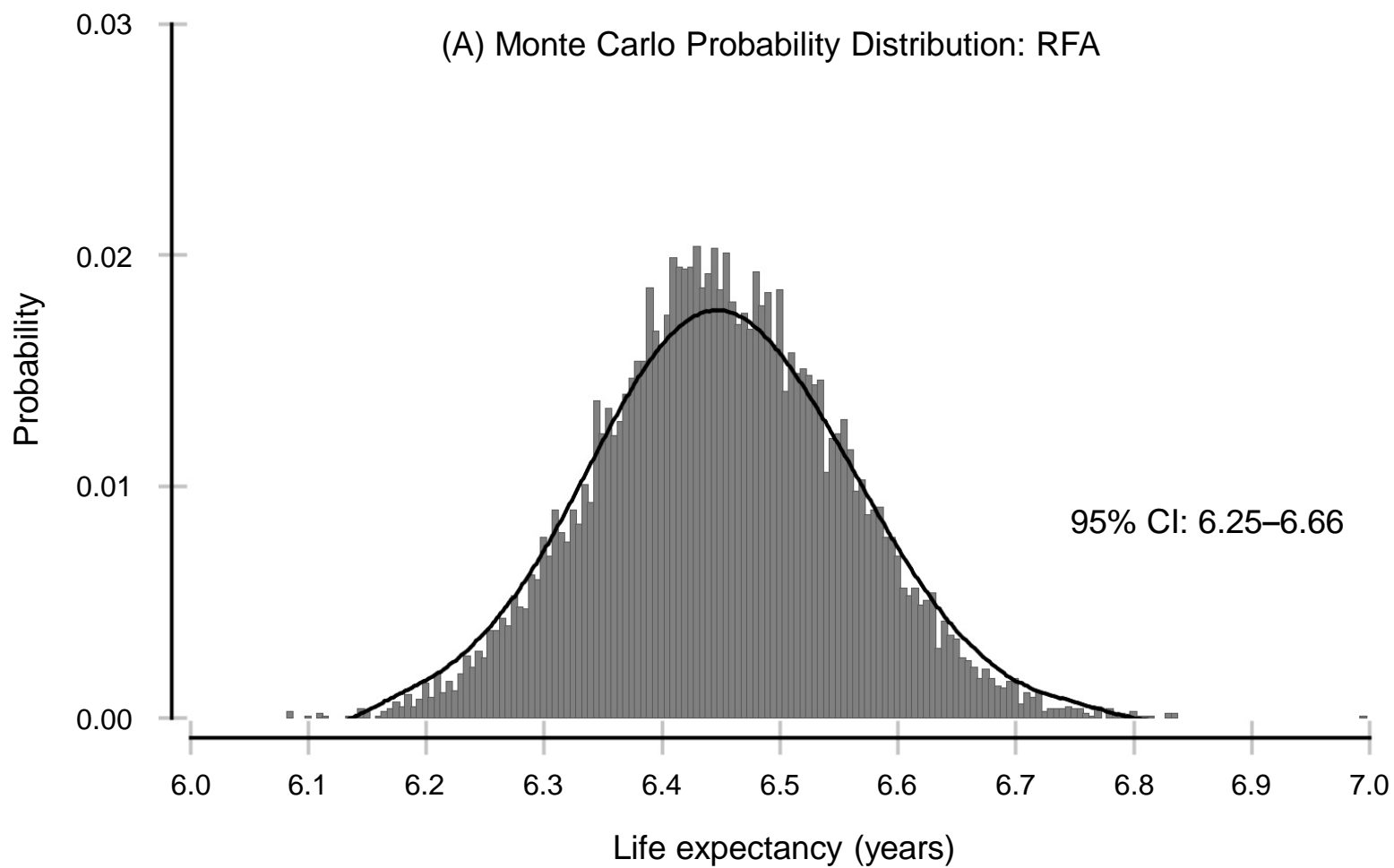

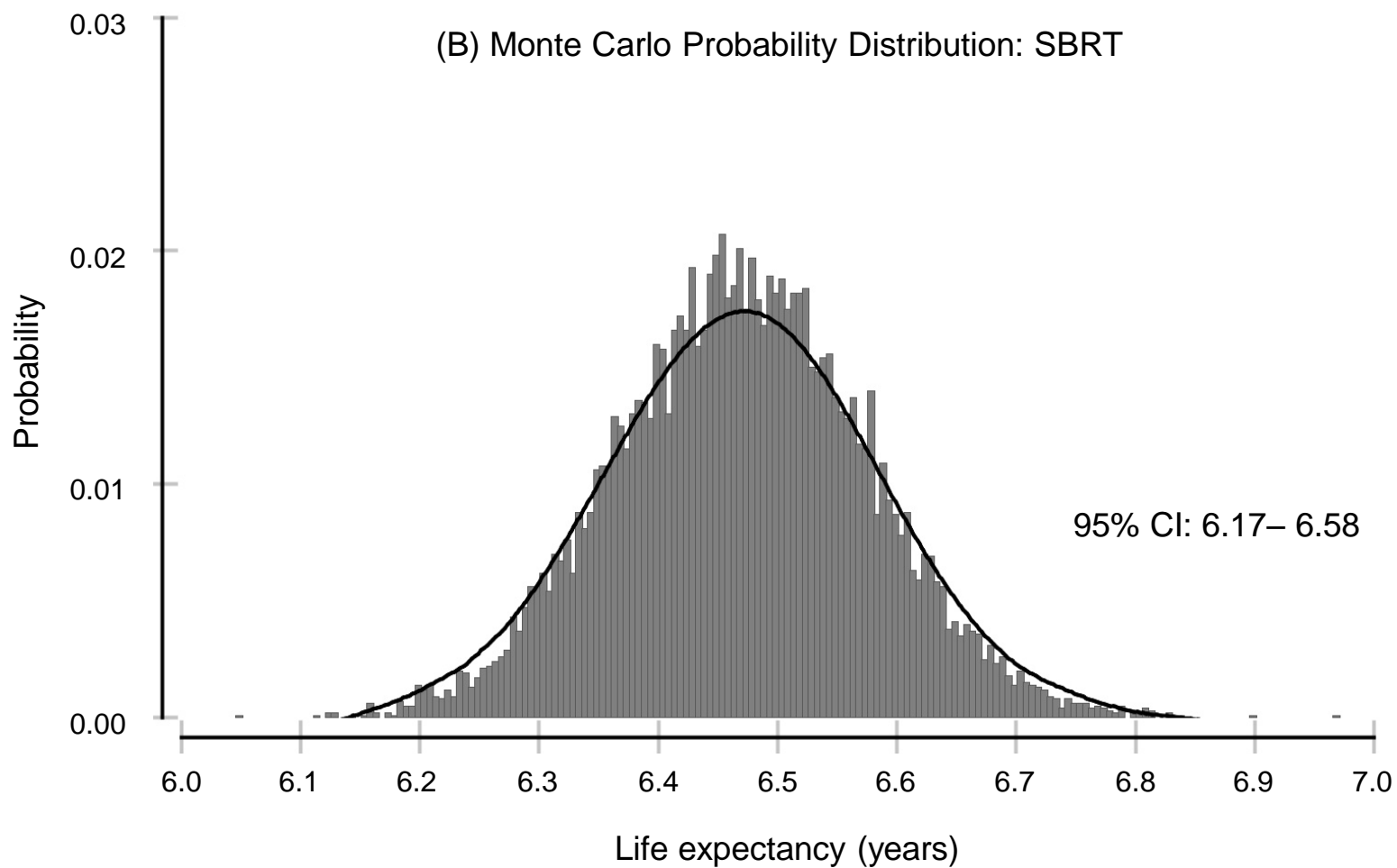

(C) Incremental outcome: RFA vs SBRT

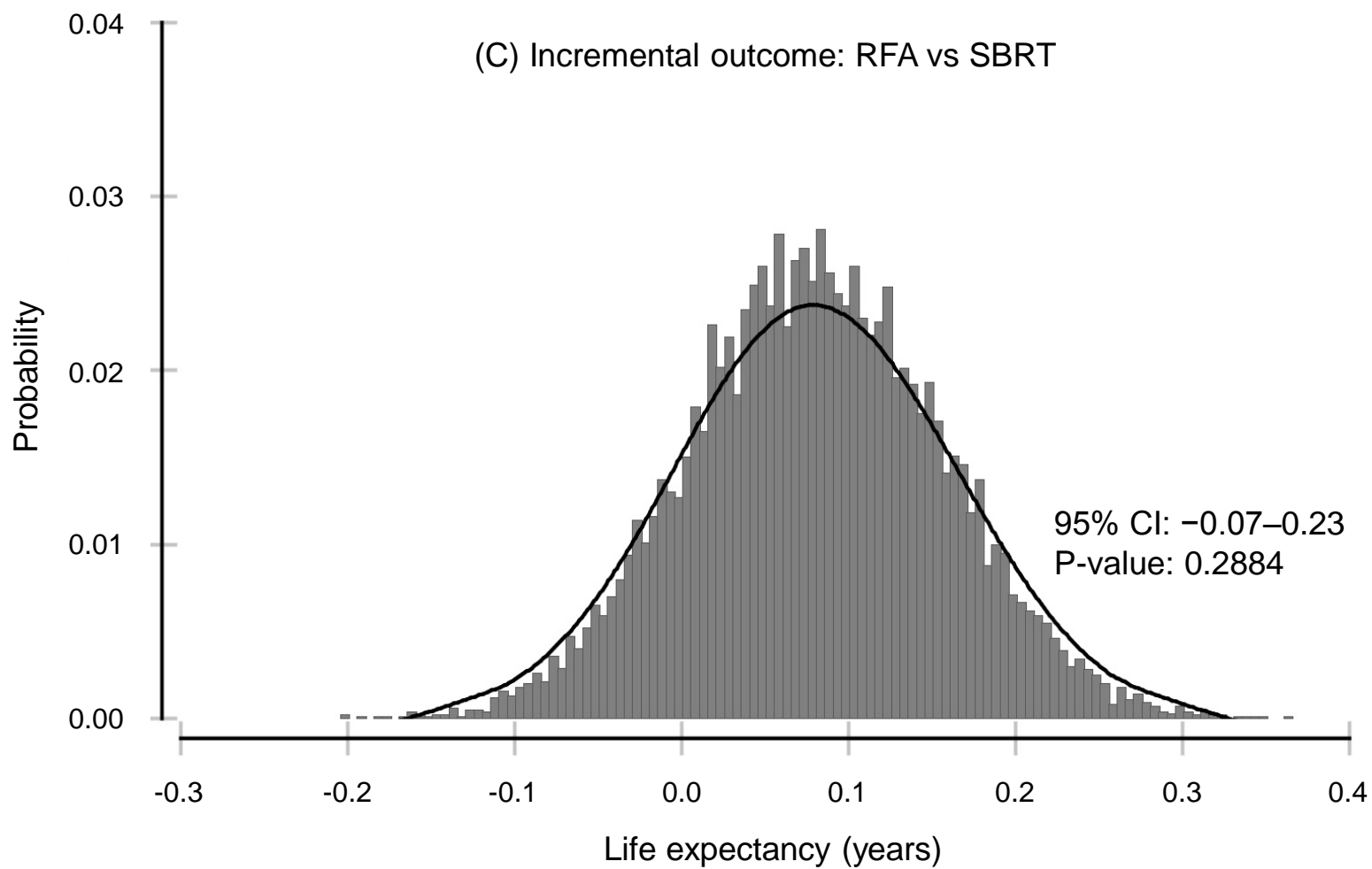

Supplement: Supplementary file 1 — Figure S1. Monte Carlo Probability Distribution: RFA Figure S2. Monte Carlo Probability Distribution: SBRT Figure S3. Incremental outcome: RFA vs. SBRT [file CAM4-5-3094-s001.pdf]
